# Latent Congruence Model to Investigate Similarity and Accuracy in Family Members' Perception: The Challenge of Cross-National and Cross-Informant Measurement (Non)Invariance

**DOI:** 10.3389/fpsyg.2021.672383

**Published:** 2021-08-11

**Authors:** Semira Tagliabue, Michela Zambelli, Angela Sorgente, Sabrina Sommer, Christian Hoellger, Heike M. Buhl, Margherita Lanz

**Affiliations:** ^1^Department of Psychology, Università Cattolica del Sacro Cuore, Brescia, Italy; ^2^Department of Psychology, Università Cattolica del Sacro Cuore, Milan, Italy; ^3^Department of Psychology, Paderborn University, Paderborn, Germany

**Keywords:** latent congruence model, measurement invariance, similarity, accuracy, cross-national, cross-informant, parent-child relationship, support exchanges

## Abstract

Several methods are available to answer questions regarding similarity and accuracy, each of which has specific properties and limitations. This study focuses on the Latent Congruence Model (LCM; Cheung, 2009), because of its capacity to deal with cross-informant measurement invariance issues. Until now, no cross-national applications of LCM are present in the literature, perhaps because of the difficulty to deal with both cross-national and cross-informant measurement issues implied by those models. This study presents a step-by-step procedure to apply LCM to dyadic cross-national research designs controlling for both cross-national and cross-informant measurement invariance. An illustrative example on parent–child support exchanges in Italy and Germany is provided. Findings help to show the different possible scenarios of partial invariance, and a discussion related to how to deal with those scenarios is provided. Future perspectives in the study of parent–child similarity and accuracy in cross-national research will be discussed.

## Introduction

More and more often, literature has suggested that, to conduct research on family relationships, it is necessary to collect data from more than one informant, although until now most family research focuses on one informant (Lanz et al., 2015). Multiple informants research allows studying interdependence among family members in many different ways, among which there is the analysis of the correspondence of perceptions of two different family members. In those cases, the level of analyses is always dyadic, but the meaning of that correspondence can be different (i.e., similarity or accuracy) depending on the position of informants in relation to the considered construct (Lanz et al., 2018).

There are many ways by which similarity or accuracy can be analyzed, some of them are able to investigate both the individual and the dyadic contribution to that correspondence (i.e., congruence), and some are also able to deal with measurement errors. This study presents in detail the Latent Congruence Model (LCM) (Cheung, 2009) as a way to test both similarity and accuracy, taking into account both cross-national and cross-informant measurement (un)equivalence. A cross-national family research on parent–child emotional support exchanges constitutes the example on which all the analyses were conducted, and Mplus syntaxes and output are provided.

### Similarity and Accuracy in Family Dyads

The congruence between scores of two informants can be conceptualized in different ways according to the position of informants in the evaluation of the construct. Indeed, as Lanz et al. (2018) underlined, informants can be embedded in the evaluation of a construct (i.e., husband evaluates his own values, wife evaluates her own values), non-embedded (i.e., father and mother evaluate values of their child), or mixed (i.e., father and child evaluate values of child). When both informants are embedded or non-embedded, the congruence of their scores is conceptualized as *similarity* (i.e., how much similar are husband and wife in their values); when informants are mixed, the *accuracy* of the score of the non-embedded informant related to the score of the embedded informant is evaluated (i.e., how much accurate is the paternal perception of values reported by the child).

Most of the family research on similarity or accuracy focuses on parent–child dyads or on romantic dyads. The similarity is, for example, investigated in studies on value similarity (Hoellger et al., 2020), personality similarity (Wang et al., 2018), or similarity in the perception of relationship conflict in parent–adolescent child dyads (Mastrotheodoros et al., 2020). Accuracy is investigated especially regarding bias in perception of characteristics of the partner (Pusch et al., 2021). Fewer studies investigate similarity and accuracy on the same construct in the same study. One exception is Decuyper et al. (2012) who studied personality similarity and accuracy in dating and married dyads.

In most of those family studies, the considered dyads are distinguishable or nonexchangeable, which means that there is at least one variable that can distinguish the two partners of the dyad. For instance, gender differentiates the two partners in heterosexual dyads; generation differentiates the two partners in parent–child dyads. In this study, we focus on distinguishable dyads, because they are more usual in family research. However, a discussion of how to deal with similarity and accuracy in exchangeable dyads (e.g., homosexual dyads, friends, colleagues) can be found in Kenny et al. (2006).

### LCM: A Way to Test Similarity and Accuracy Taking Into Account the Measurement Model

Among the most intuitive approaches to the study on similarity and accuracy there is the creation of dyadic indexes such as the mean-level difference scores (e.g., algebraic, absolute, and squared difference) and the profile similarity indexes (e.g., sum of absolute/squared difference, profile correlations) (Edwards, 1994; Lanz et al., 2018). However, research that computes dyadic indexes usually does not consider the measurement model that informs of the scores used to compute those dyadic indexes. Since most of the instruments chosen to evaluate family relationships and functioning are based on the classical test theory, computing congruence between two informants also considering the measurement model could increase the interpretability of findings related to dyadic congruence.

Some recent reviews of the literature (De Los Reyes et al., 2019a,b) supported the fact that discrepancies of informants are meaningful and related to criterion validity variables (De Los Reyes et al., 2015); thus, they are not mainly explained by measurement confounds because of the way informants are using instruments to evaluate targets. However, other researchers (Russell et al., 2016) still underline the importance of testing models based on equivalent cross-informant measurement models to have more precise estimates of congruence. Moreover, dyadic research underlines the importance of testing dyadic measurement invariance in dyadic research designs (Tagliabue and Lanz, 2014; Claxton et al., 2015; Sakaluk et al., 2021), and some research findings revealed that measurement models tested with CFA applied to dyads are not always fully invariant across dyad members. For instance, DeLuca et al. (2019) found partial metric invariance and/or partial scalar invariance for Adult Self Report (ASR) and the Adult Behavior Checklist's (ABCL) dimensions.

To deal with measurement model, SEM models that allow both to test the measurement model of scores of dyadic partners, and to model similarity and accuracy at a latent level have been proposed. The measurement model (often tested through CFA) is related to the way items are linked with latent variables, and such item-factor relationship is described by different parameters: factor loadings, intercepts, and residuals. Testing dyadic measurement invariance means to verify that each of those parameters is equivalent across the two informants (i.e., parent and child). In particular, measurement invariance levels are configural, metric, strong, and strict (Steenkamp and Baumgartner, 1998; Claxton et al., 2015). Configural invariance level means that the confirmatory measurement model fits well for both parents and children, although all the parameters (factor loadings, intercepts, residuals) are free to be different in parents and children. When metric invariance is found, it means that the meaning of the latent factor is equal for both partners; indeed, technically speaking, factor loadings are constrained to be equal for parents and children. Strong invariance means that the mean of the different items is the same for parents and children. Finally, strict invariance means that the measurement error amount is the same for both partners (and it also means that the shared variance of each item is the same for both parents and children). It is important to evaluate and keep measurement model and measurement invariance into consideration when investigating and interpreting similarity or accuracy (Edwards, 2009).

**Figure 1:**
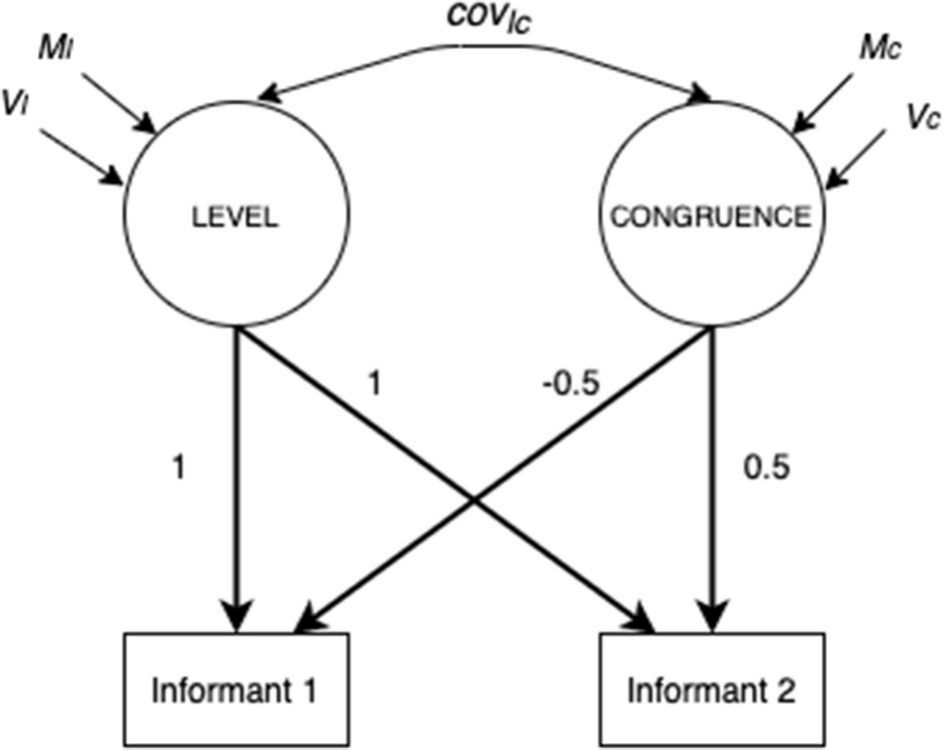
Simplest LCM on observed variables (Figure adapted from Cheung, 2009, p. 9).

Among the SEM models able to test similarity and/or accuracy in dyadic perceptions, the Latent Congruence Model (LCM; Cheung, 2009) allows testing both similarity and accuracy within the same framework checking for cross-informant measurement invariance. LCM is a specific SEM model in which the unit of analysis is the dyad. The simplest LCM involves two observed variables on the same construct from two informants (i.e., perception of father and child of their quality of communication). Figure 1 shows the simplest LCM. The variance of the two observed variables is disentangled into two factors, the first is called LEVEL and the second is called CONGRUENCE. LEVEL is the mean of the two observed variables, and it is estimated fixing to 1 the factor loadings of the two observed variables on the LEVEL factor. CONGRUENCE is the difference between the two observed variables, and it is estimated by fixing to 0.5 or −0.5 the two factor loadings of the observed variables on the CONGRUENCE factor, so that difference is equally divided for the two observed variables and the direction of the difference (i.e., which is the higher or lower score) can be interpreted. The mean and variance of the LEVEL factor are the grand mean and the variance of the mean scores of all the dyads, whereas the mean and the variance of the CONGRUENCE factor are respectively the average difference between scores of the two dyadic members and its variability across dyads.

The simplest LCM is not able to work on measurement model, thus a more complex LCM, called item-level LCM, was proposed by Cheung (2009). In that model, instead of the two observed variables, there are two latent variables, measured by items filled in by the two informants. Thus, LCM becomes a two-level SEM. First-level latent variables represent the perception of each partner, so it is possible to test cross-informant measurement invariance putting equivalence constraints across partners. Second-level variables are LEVEL and CONGRUENCE already explained above. It is important to note that the CONGRUENCE variable is a general label indicating the difference between the two perceptions, but, according to the variables and informants considered, it could be interpreted as lack of similarity (L_SIM), or lack of accuracy (L_ACC). LCM focused on lack of similarity and LCM focused on lack of accuracy are slightly different, and the details of those differences are presented in the data analysis section.

LCM was first proposed within organizational psychology (Cheung, 2009), an area of psychology that frequently uses congruence or discrepancy scores. However, since then, LCM has been often used in family research too. For instance, Schaffhuser et al. (2016) used LCM to investigate discrepancy in dyadic perceptions of personality traits within romantic couples, also analyzing actor and partner association with relationship satisfaction. Also, Reifman and Niehuis (2018) and Mastrotheodoros et al. (2020) used LCM within longitudinal designs, testing measurement invariance in both dyadic and longitudinal aspects and then evaluating how discrepancy in couple or parent–child relationships changes overtime.

### Cross-Cultural Comparisons on Similarity and Accuracy

Recently, a meta-analysis (De Los Reyes et al., 2019b) revealed that congruence is related to cultural characteristics. Moreover, as Rescorla (2016) underlined, there is a need for cross-cultural direct comparisons regarding parent–child discrepancies. Very few studies directly compared parent–child discrepancy across nations, and the ones that did, as for example Rescorla et al. (2013), did not test cross-cultural measurement invariance. We also did not find applications of LCM to cross-cultural or cross-national studies. One possible explanation is the lack of discussion about how to keep into consideration at the same time cross-informant and cross-national (or cross-cultural) measurement invariance. Indeed, single sample applications of LCM show how to test cross-informant measurement invariance when estimating second-order level and congruence variables. However, in cross-national (or cross-cultural) designs, a preliminary step is needed, which is verifying the presence of cross-national (or cross-cultural) measurement invariance before testing the cross-informant one and applying the whole LCM. This study proposes a step-by-step guide to conduct those kinds of analyses and interpret cross-national differences in similarity and accuracy within parent–child dyads.

### The Study

There are three aims that guided this study: (1) to investigate parent–adult child similarity and accuracy through LCM using illustrative data on given or received emotional support in German and Italian samples; (2) to discuss how to test cross-national and cross-informant measurement invariance within LCM framework; and (3) to compare findings controlling, or not, for cross-national and cross-informant measurement invariance, and discuss possible interpretations of findings.

## Materials and Methods

Illustrative data are composed of 195 Italian and 417 German family triads (father, mother, adult child). Young-adult children (43.7% males) were aged between 25 and 35 (*M* = 29.66; *SD* = 3.07), mothers were aged between 41 and 87 (*M* = 58.07; *SD* = 6.24) and fathers between 41 and 87 (*M* = 60.78; *SD* = 7.19).

The sample for this research derives from the “Interdependence in adult child-parent relationships” project funded by the German Research Foundation (DFG). The study was conducted between 2016 and 2017. In response to advertisements, the participants contacted the project team and got the questionnaires *via* mail (Germany) or directly by hand from the research team (Italy). The participation was voluntary, and the participants could quit the study at any time. Data were collected confidentially and anonymously. The data of adult children, fathers, and mothers were matched by personal codes, but the data were not attributable to individual participants.

Five hundred eighty-four mother–child dyads (395 German and 189 Italian) and 599 father–child dyads (406 German and 193 Italian) were included in data analyses for the estimation of similarity and accuracy LCMs.

The German and the Italian sub-samples were mostly comparable with respect to sociodemographic variables for each of the three perceivers (Table 1). However, some differences were found between the two. Specifically, German children seem to have reached some adulthood markers more than Italian ones (only 18.8% German children cohabitate with parents; and 26.6% of them are parents).

**Table 1:** Descriptive statistics and cross-national comparison in Germany and Italy.

		**Descriptive statistics**		**Cross-nations comparison**	
**Perceiver**		**Germany**	**Italy**	***t*/χ^2^ (df)**	***p*-value**
**Age (Mean, SD)**	Mother	58.9 (5.33)	57.7 (6.60)	−2.19 (416.34)	0.029
	Father	62.3 (6.04)	60.1 (7.56)	−3.38 (366.32)	<0.001
	Child	29.4 (3.01)	29.8 (3.07)	1.28 (364.82)	0.202
**Sex (%, N)**	Child (Males)	44.8% (82)	43.2% (158)	0.133 (1)	0.715
**Marital status (%, N)**					
Single	Mother	3.9% (14)	1.7% (3)	2.64 (3)	0.450
Married		81.5% (296)	83.1% (148)		
Divorced		11.6% (42)	10.7% (19)		
Widowed		3% (11)	4.5% (8)		
Single	Father	3.4% (12)	1.3% (2)	2.02 (3)	0.568
Married		86.1% (303)	87.3% (138)		
Divorced		9.1% (32)	9.5% (15)		
Widowed		1.4% (5)	1.9% (3)		
Single	Child	73% (267)	81.4% (149)	5.76 (2)	0.056
Married		26% (95)	16.9% (31)		
Divorced		-	-		
Widowed		1.1% (4)	1.6% (3)		
**Children (%, N)**	Child	Yes: 26.6% (97)	16.5% (30)	6.94 (1)	0.008
**Cohabitation with parents (%, N)**	Child	Yes: 18.8% (69)	45.9% (99)	44.66 (1)	<0.001
**Occupation child (%, N)**					
Students	Child	27% (94)	13.8% (23)	18.80 (4)	<0.001
Training student		2.6% (9)	7.8% (13)		
Worker		50.9% (177)	61.7% (103)		
Student-worker		15.8% (55)	13.2% (22)		
Not student, not worker		3.7% (13)	3.6% (6)		
**Occupation parents (%, N)**					
Worker	Mother	54.6% (197)	49.4% (84)	36.13 (4)	<0.001
Occasional Job		13% (47)	1.2% (2)		
House-wife		12.5% (45)	24.1% (41)		
Looking for a job		4.4% (16)	1.2% (2)		
Retired		15.5% (56)	24.1% (41)		
Worker	Father	66.8% (231)	60.9% (92)	7.70 (4)	0.103
Occasional Job		1.4% (5)	0.7% (1)		
House-husband		1.4% (5)	0.7% (1)		
Looking for a job		4% (14)	1.3% (2)		
Retired		26.3% (91)	36.4% (55)		

*t: statistical value of Student's t-test; χ^2^: statistical value of Pearson chi-square*.

### Instrument

Each family member (father, mother, adult child) filled in the nine-item emotional support subscale from the Given and received support scale (Sommer and Buhl, 2018). The instrument consisted of two items of instruments used in the *Panel Analysis of Intimate Relationships and Family Dynamics* (Pairfam; Thönnissen et al., unpublished^1^) and seven *ad hoc*-developed items. The instruction and items composing the instrument are presented in Appendix A in Supplementary Material. The items were translated from German to English and Italian and then back-translated for control by native speakers of German and Italian.

Each family member answered the nine items referring to the other two members, and emotional support was measured separately for given and received support. Specifically, in this study, young adult children provided their perception of giving support to mother (ω = 0.901) and father (ω = 0.906) and of receiving support from mother (ω = 0.931) and father (ω = 0.942) in the past 12 months, whereas mothers and fathers provided their perceptions of giving support to their child (respectively ω = 0.925 and 0.925) and of receiving support from him/her (respectively ω = 0.937 and 0.937) in the past 12 months. Each participant was asked for the given support “What kinds of help did you give to your child/father/mother in the last 12 months?” and for the received support “What kinds of help did you receive from your child/father/mother in the last 12 months?”; the participants rated items such as “Advice regarding personal problems (item 1)” or “Talk about my/their worries and troubles (item 8)” referring to each of the other family members. Items were rated on a 5-point Likert scale from 1 (never) to 5 (always). The scale we used was highly reliable even when the composite reliability (ω) was estimated separately for each country (see Supplementary Material).

## Data Analyses

### Missingness Analysis and Outliers

Missing data were handled in Mplus *via* the full information maximum likelihood (FIML) method. We evaluated missingness percentage at dyadic, respondent, and item level because of family data (Tagliabue and Donato, 2015). Then, the missingness mechanism was evaluated by Little's MCAR test. Multivariate outliers were analyzed by Mahalanobis Distance based on chi-square distribution significant for *p* < 0.001 (Tabachnick and Fidell, 2013).

### Latent Congruence Model (Aim 1)

In the illustrative example, we asked parent–child dyads to evaluate given and received emotional support within their relationship. For instance, the mother evaluates how much emotional support she is giving to her adult child and how much emotional support she is receiving from that child. At the same time, the adult child evaluates how much emotional support he/she is giving to his/her mother and how much emotional support he/she is receiving from her.

When LCM is applied to perceptions of mother and adult child of given emotional support, dyadic similarity regarding the perception of given emotional support is evaluated; the same for perception of mother and adult child of received emotional support in their relationship. When LCM is applied, on one side, to perception of mothers of given emotional support to the adult child and perception of the adult child of received emotional support from the mother, and, on the other side, to perception of mothers of received emotional support from adult child and perception of adult child of given emotional support to mother, accuracy in exchanged emotional support is evaluated.

**Figure 2:**
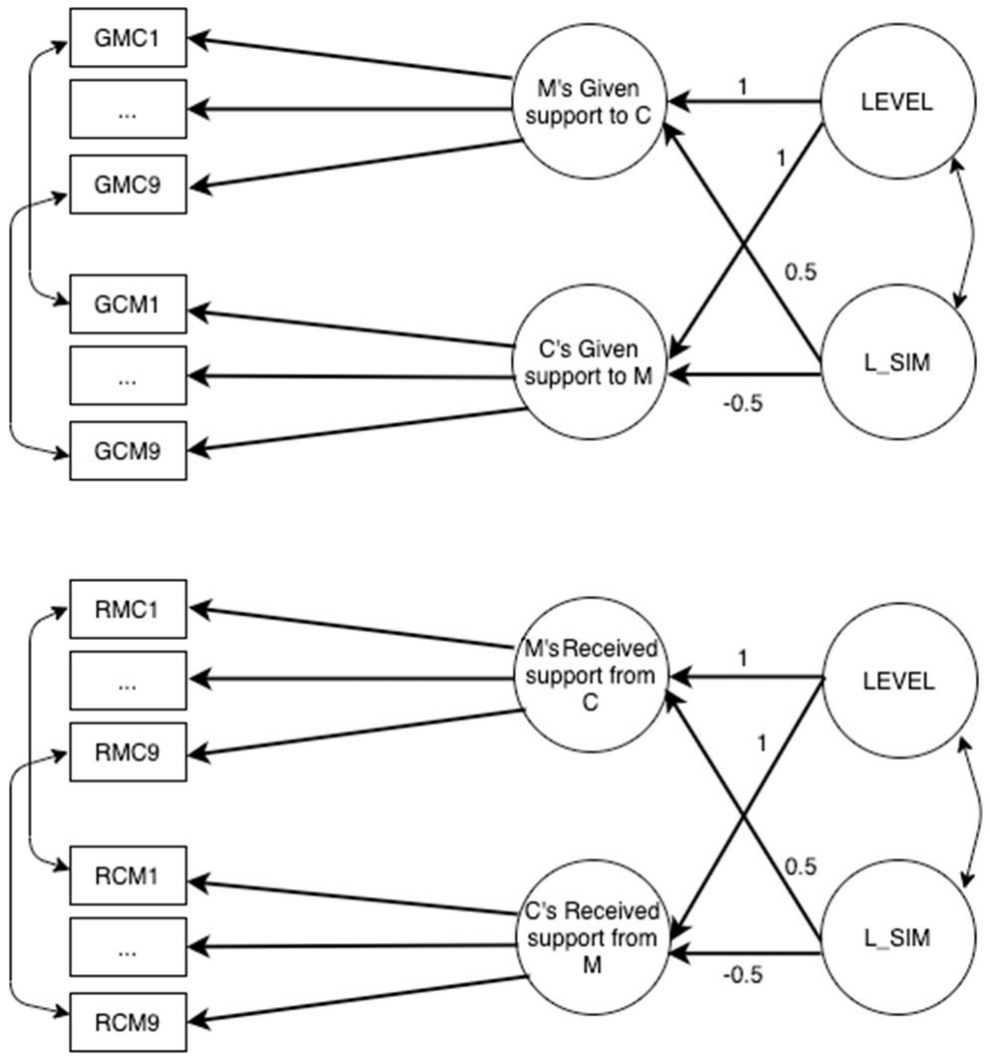
Similarity LCM referred to mother–child given support M, mother; C, child. GMC1–GMC9, items assessing the support given by the mother; GCM1–GCM9, items assessing the support given by the child; LEVEL, average (mean) level of the mother and child perceptions of given support (second-order factor); L_SIM, lack of similarity (second-order factor).

**Figure 3:**
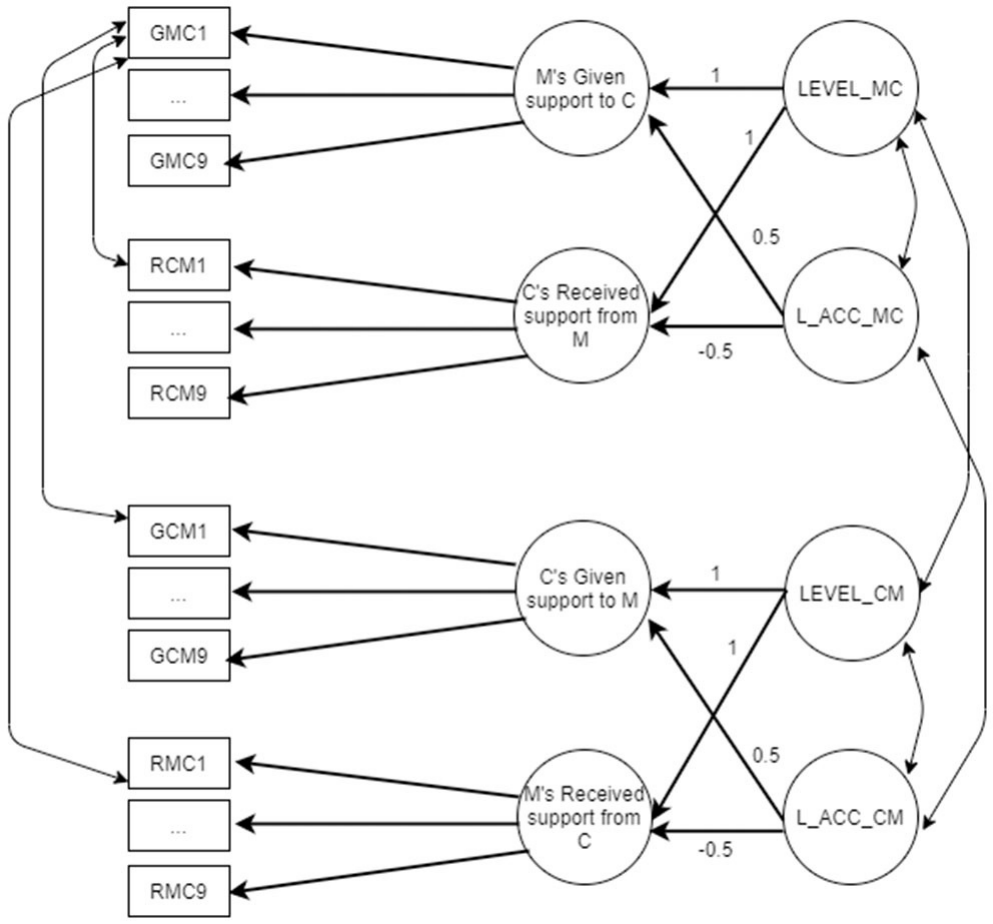
Accuracy LCM referred to mother–child relationship M, mother; C, child. GMC1–GMC9, items assessing the support given by the mother; GCM1–GCM9, items assessing the support given by the child; RMC1–RMC9, items assessing the support received by the mother; RCM1–RCM9, items assessing the support received by the child; LEVEL_MC, average (mean) level of mother and child perceptions for the downward support exchange (second-order factor); LEVEL_CM, average (mean) level of mother and child perceptions for the upward support exchange (second-order factor); L_ACC_MC, lack of accuracy for the downward support exchanges (second-order factor); L_ACC_CM, lack of accuracy for the upward support exchanges (second-order factor).

Two different latent congruence models (LCMs) were tested, one for similarity and the other for accuracy, separately for the German and Italian samples. In all LCMs, constraints related to full cross-informant measurement invariance were inserted. Figure 2 presents the similarity LCM, whereas Figure 3 presents the accuracy one. All the models were tested using Mplus (RRID:SCR_015578).

Similarity LCM is applied separately for given and received support (Figure 2), and for mother–child and father–child dyads (for a total of four models). In each similarity LCM, there are two second-order factors: LEVEL is estimated constraining to 1 factor loadings of first-order factors, whereas lack of similarity factor (L_SIM) is estimated constraining to 0.5 the first-order factor of parent and to the −0.5 the first-order factor of child. Item residuals were correlated across the two informants, so for instance, residual of item 1 referring to perception of mother of given support to child was correlated to residual of item 1 referring to perception of child of given support to mother, and the same was done for all residuals of the items. Figure 2 shows only covariances referred to item 1, but the same is done for all the nine items.

Figure 3 presents the accuracy LCM. Accuracy is evaluated separately for mother–child and father–child dyads. Items measuring given and received support both from mother and child are included in the same LCM. Each LCM includes four first-order factors (support given by the mother, support received by the child, support given by the child, and support received by the mother) and four second-order factors: LEVEL and lack of accuracy (L_ACC) factors for the support given by the mother and received by the child (also called downward support exchanges, Kim et al., 2014), as well as LEVEL and lack of accuracy (L_ACC) factors for the support given by the child and received by the mother (also called upward support exchanges, Kim et al., 2014). As happened for similarity LCM, LEVEL is estimated constraining to 1 factor loadings of first-order factors, whereas lack of accuracy factor (L_ACC) is estimated constraining to 0.5 the given support first-order factor and to −0.5 the received support first-order factor. Covariances among item residuals are estimated across items with the same wording. Figure 3 shows only covariances referred to item 1, but the same is done for all the nine items.

For all the LCMs, the goodness of model fit was evaluated using the following indices: chi-square test (χ^2^), which indicates good fit when it is not significant (*p* > 0.05); root mean squared error of approximation (RMSEA) and the standardized root mean square residual (SRMR), which indicate good fit when lower than 0.08; and the comparative fit index (CFI), which indicates good fit when higher than 0.9 (Marsh et al., 2004). At the following link https://osf.io/t54yf/?view_only=a10011bc93f24cc6ad8bb6ea4cbe6a0a Mplus LCM input and output referred to similarity and accuracy models of both relationships are available.

### Cross-National and Cross-Informant Invariance (Aim 2)

A multigroup model was applied, first testing if a configural first-order model fits well in the two samples (German and Italian ones). This means that given and received support first-order latent variables were estimated, without adding second-order latent variables of LCM. Measurement invariance, testing if factor loadings of the items (metric invariance), intercepts (scalar invariance), and residuals (strict invariance) were equivalent across groups, was verified first across nations (Germany vs. Italy) and then across informants (parent vs. child). The reason for that choice is rooted in the construct we are measuring. Similarity (or accuracy) is measured starting from the individual perception of the relationship by each informant. When cross-national comparisons are done, we need to verify first that there is an equivalent measurement model of the same construct evaluated by the same informant in the two nations, and, in a second step, whether there is also cross-informant measurement invariance.

To compare nested models, we compared chi-square, RMSEA, and CFI values across models. A significant chi-square difference (*p* < 0.05), a decrease in the CFI higher than 0.1, and an increase in the RMSEA equal to or higher than 0.015 were considered indicating a substantial decrease in model fit (Chen, 2007). When the difference was detected only by the chi-square test, it was not considered relevant as it is well-known that the chi-square significance is also affected by other factors such as the sample size (Iacobucci, 2010). At the following link https://osf.io/t54yf/?view_only=a10011bc93f24cc6ad8bb6ea4cbe6a0a Mplus input and output files produced to test the cross-national and cross-informant measurement invariance referred to similarity and accuracy models of both relationships are available.

### Cross-National and Cross-Informant Measurement Invariance Within LCM (Aim 3)

Multigroup LCM was tested first without applying cross-national and cross-informant constraints, and, second, adding the cross-national and cross-informant measurement invariance constraints tested in the aim 2 section. This was done to demonstrate whether differences in cross-national parent–child similarity and accuracy could be due to measurement non-invariance reasons. Equality constraints were put on LEVEL and lack of similarity or accuracy (L_SIM or L_ACC) second-order factors of Italian and German samples to compare parent–child congruence scores (variance and mean) in the two nations. At the following link https://osf.io/t54yf/?view_only=a10011bc93f24cc6ad8bb6ea4cbe6a0a Mplus input and output files produced to test similarity and accuracy LCM of both relationships are available. Moreover, a table with all the different steps of the analyses is presented in Appendix B in Supplementary Material.

## Results

### Missingness Analysis and Outliers

Missingness analysis was conducted on the complete dataset made of 612 families (Germany and Italy). The findings are presented in Table 2. Overall, the missingness rates were similar for Germany and Italy. Specifically, missing data at the dyad level ranged between 1.5 (Italian father–child dyad) and 2.9% (German mother–child and father–child dyad). Missing data at the respondent level ranged between 1.5 (Italian mothers) and 12.3% (Italian fathers). Missingness at the item level never exceeded 10% for each version of the received and given support scale. Regarding missingness mechanism, Little's MCAR test indicates that data were distributed randomly for the father–child relationship for both Germany [χ^2^(1,163) = 1224.02; *p* = 0.104] and Italy [χ^2^(645) = 609.71; *p* = 0.837]; while a different mechanism of missingness was revealed for the mother–child relationship, as missing data were distributed randomly for the Germany group [χ^2^(1,154) = 1,224.91; *p* = 0.072], and non-randomly for the Italian group [χ^2^(707) = 793.79; *p* = 0.013]. Multivariate outliers for the father–child dyad (11 German dyads, two Italian dyads) and for the mother–child dyad (22 German dyads, six Italian dyads) were excluded, and ML was used as estimator in subsequent analysis (similarity and accuracy models).

**Table 2:** Missingness rates at the dyad, respondent, and item level for the German and Italian samples.

**Family/dyad member**	**Missingness at the dyad level**		**Missingness at the respondent level**		**Missingness at the item level**			
					**Given**		**Received**	
	**Germany**	**Italy**	**Germany**	**Italy**	**Germany**	**Italy**	**Germany**	**Italy**
Father-child	2.9%	1.5%	5.8%	12.3%	2.3–3.4%	0.6–4.4%	4.8–5.9%	2.5–3.1%
Mother-child	2.9%	2.1%	2.4%	1.5%	1.1–2.4%	1.7–3.9%	3.0–4.1%	3.4–5.6%
Child-mother	2.9%	2.1%	5.5%	2.6%	1.9–2.7%	2.2–4.9%	1.9–2.7%	2.2–3.3%
Child-father	2.9%	1.5%			4.9–5.4%	6–8.7%	5.4–5.9%	6–6.6%

### Similarity and Accuracy

The findings will be presented separately for similarity and accuracy. Within each section, findings related to the three aims will be described, following a step-by-step structure. For the sake of brevity, we present only results referring to the mother–child relationship. Results referring to the father–child relationship are reported in Supplementary Material.

### Similarity

#### LCM (Aim 1)

To realize aim 1, we run two LCMs separately for each nation. In particular, we tested two similarity models for the mother–child relationship, the *given support similarity model*, which allows comparing perception of the child of given support to the mother with perception of the mother of given support to her child, and the *received support similarity model*, which manages to compare perception of the child of received support from the mother with perception of the mother of received support from her child.

Each LCM included two first-order factors (support given/received by the child and support given/received by the mother), and two second-order factors (LEVEL factor and L_SIM factor for the support given/received by the child and the mother). In each model, we imposed cross-informant constraints, i.e., factor loadings of items, intercept, and residuals were imposed to be equivalent for mother and child reports when the two informants were evaluating the support they gave/received to each other (e.g., equivalence between the support given by the mother and given by the child). These models were run to estimate the amount of (lack of) similarity between the two informants. In other words, we estimated from these models the mean and the variance of the L_SIM second-order factor(s), whereas we constrained the mean of LEVEL to be 0, and we estimated its variance.

Both models (given and received support similarity models) run on the German sample had sufficient fit indices [given support: χ^2^(150) = 497.76; *p* < 0.001; RMSEA = 0.078 (0.071 0.086); CFI = 0.912; SRMR = 0.094; received support: χ^2^(150) = 509.20; *p* < 0.001; RMSEA = 0.079 (0.072 0.087); CFI = 0.925; SRMR = 0.068]. The lack of similarity for the given support between mother and child was 0.096^2^ (*p* = 0.022) [variance = 0.468 (*p* < 0.001)], and the variance of the LEVEL factor was 0.460 (*p* < 0.001); while for the received support, the lack of similarity between mother and child was −0.235 (*p* < 0.001) [variance = 0.580 (*p* < 0.001)], and the variance of the LEVEL factor was 0.546 (*p* < 0.001). The same models were run for the Italian sample obtaining worse fit indices for both models because of the smaller sample size [given support: χ^2^(150) = 444.81; *p* < 0.001; RMSEA = 0.104 (0.093, 0.115); CFI = 0.846; SRMR = 0.103; received support: χ^2^(150) = 445.26; *p* < 0.001; RMSEA = 0.104 (0.093, 0.115); CFI = 0.890; SRMR = 0.082]. In this nation, the lack of similarity was on average 0.261 (*p* < 0.001) [variance = 0.425 (*p* < 0.001)] for the given support, and the variance of the LEVEL factor for the given support was 0.430 (*p* < 0.001); while for the received support, the lack of similarity was −0.112 (*p* = 0.097) [variance = 0.676 (*p* < 0.001)] and the variance of the LEVEL factor was 0.522 (*p* < 0.001). In both nations, mother perceives to give more and receive less support than the child. Similarity models referred to father–child relationship showed that, only in Italy, father perceives to give more support than child. On the contrary, similarity in received support (both nations) and similarity in given support (only Germany) was found in father–child dyads (see Supplementary Material).

#### Cross-National and Cross-Informant Invariance (Aim 2)

As the measurement invariance issue concerns only the first-order factors, we run the two models (*given support similarity model* and *received support similarity model*) presented in LCM (aim 1) but the second-order factors were removed. Models were not applied separately to German and Italian samples, but we tested them within a multigroup model. Furthermore, as required to fully test the measurement invariance of each item composing the scale, we fixed to 0 the means of all first-order factors and to 1 all first-order factor variances of the first group (Germany), while they were freely estimated for the second group (Italy). All factor loadings of both groups were freely estimated.

Regarding the *given support similarity model*, the configural model presented good model fit indices (see Table 3). We then proceeded testing cross-national measurement invariance by verifying if items reported by the same informant (e.g., child) thinking of giving support to the other informant (e.g., mother) had equivalent factor loadings (metric cross-national invariance), intercepts (scalar cross-national invariance), and residuals (strict cross-national invariance) across Germany and Italy.

**Table 3:** Cross-national and cross-informant invariance of the similarity model for the given support mother–child relationship.

**Model**	**χ^2^**	***p***	***df***	**RMSEA**	**RMSEA (90% CI)**	**CFI**	**SRMR**	**Δχ^2^**	***Δdf***	***p***	**ΔCFI**	**ΔRMSEA**
Configural	593.93	<0.001	250	0.070	(0.063 0.077)	0.941	0.050					
**Cross-national invariance**												
Metric	647.35	<0.001	266	0.071	(0.064 0.078)	0.935	0.074	53.42	16	0.086	−0.006	0.001
Scalar	915.87	<0.001	282	0.089	(0.083 0.096)	0.892	0.113	268.51	16	<0.001	−0.043	0.018
Unconstrained to be equal: GCM06, GMC06, GCM03, GMC03, GCM04	705.69	<0.001	277	0.074	(0.067 0.081)	0.927	0.077	58.33	11	<0.001	−0.008	0.003
Strict	730.85	<0.001	290	0.074	(0.067 0.080)	0.925	0.081	25.16	14	0.022	−0.002	0.000
**Cross-informant invariance**												
Metric	759.53	<0.001	298	0.074	(0.068 0.081)	0.921	0.089	28.68	8	<0.001	−0.004	0.000
Scalar	862.30	<0.001	308	0.074	(0.074 0.086)	0.905	0.099	102.77	10	<0.001	−0.016	0.006
Unconstrained to be equal: IT: GCM01; GE: GMC03;GCM03	805.79	<0.001	306	0.078	(0.070 0.083)	0.915	0.095	46.25	8	<0.001	−0.006	0.002
Strict	908.97	<0.001	315	0.076	(0.076 0.088)	0.898	0.109	103.18	9	<0.001	−0.017	0.006
Unconstrained to be equal: GE: GMC05, GMC07; IT: GMC07	858.29	<0.001	312	0.079	(0.073 0.085)	0.907	0.099	52.51	6	<0.001	−0.008	0.003

*χ^2^, chi-square test; df, degree of freedom; RMSEA, root mean square error of approximation; CI, confidence interval; CFI, comparative fit index; SRMR, standardized root mean square residual. For each released item: G, given support; MC, mother reports on child; CM, child reports on mother; 01–09: item's number, thus, for example, GCM01 means item 1 of given support when child reports on mother; GE/IT, Germany/Italy*.

For the two factors (e.g., support given by the child, support given by the mother) included in the model, we found that their items had equivalent factor loadings across nations (full metric invariance). Instead, regarding the scalar invariance, we found that item 6 (“Offered to conduct a conversation”) and item 3 (“Conducted conversations concerning my child/mother personal topics”) worked differently across nations for each of the two factors, while item 4 (“Conducted conversations concerning my mother daily issues”) worked differently across nations only when the child refers to the mother. In particular, item 3 had higher intercepts in Germany than in Italy both when the informant was the mother (3.72 vs. 3.38) as well as the child (3.82 vs. 3.41), the same for item 4 when the child was the informant (4.14 vs. 3.87). On the contrary, item 6 showed higher intercepts in Italy than in Germany both when the informant was the mother (3.76 vs. 3.43), as well as the child (4.04 vs. 3.28). In other words, German mothers and children tend to score item 3 higher than Italians, German children tend to score item 4 higher than Italians, while Italian mothers and children tend to score item 6 higher than Germans.

Keeping into account the three non-invariant intercepts, we proceeded testing the strict cross-national invariance. Tested residuals (all except residuals of non-invariant items from the scalar invariance) were equivalent across nations. We can conclude that given support factors of mother and child have the same meaning within the two nations (metric cross-national invariance), but their latent means are not fully comparable across groups because some intercepts differ across the two nations.

Maintaining in the model the cross-national invariance constraints, we proceeded testing the measurement invariance across informants. In other words, we verified if the instrument works equivalently across the two informants who are reporting about giving support to each other.

We imposed cross-informant constraints between items in which the child reported the given support to the mother, and items in which the mother reported the support she gave to her child. Constraints were imposed starting from factor loadings of items (metric cross-informant invariance) and proceeding with intercepts of items (scalar cross-informant invariance) and residuals (strict cross-informant invariance). We found equivalent factor loadings across the two informants (full metric invariance). Instead, we found that item 1 (“Advice regarding personal problems”) had different intercepts across informants for the Italian group, while item 3 (“Conducted conversations concerning my child/mother personal topics”) had different intercepts across informants for the Germany group. Specifically, Italian mothers tend to score higher in item 1 (3.19 vs. 2.84) than their children, while German children tend to score higher in item 3 (3.82 vs. 3.56) than their mothers.

We proceeded by testing the equivalence of residuals across informants, finding that residuals of items 5 and 7 were different across informants for the German group, while only the residual of item 7 was different across informants for the Italian group. Specifically, the residual variances were higher when the child was the informant both for item 7 in the Italian group (0.413 vs. 0.212), and for item 5 (0.311 vs. 0.180) and item 7 (0.413 vs. 0.252) in the German group. These findings revealed that, also from a cross-informant point of view, measurement model is not fully invariant, showing that if a lack of similarity will be found, it could be also because of cross-informant differences in intercepts.

We followed exactly the same procedure for the *received support similarity model* (see Table 4). In this case, we found a partial metric invariance within the cross-national comparison, as the factor loading of item 6 of the child reporting the received support from the mother was higher for the German group (0.885 vs. 0.578). In other words, item 6 (“Offered to conduct a conversation”) is much more representative of the support received by mothers for German children compared with Italians. Intercepts and residuals were equivalent across nations except for the intercept of item 6 when reported by the mother, as it was higher for Italy (3.67 vs. 3.24). We can conclude that given support factors of mother and child have only partially the same meaning within the two nations (partial metric cross-national invariance), and also their latent means are not fully comparable across groups because some intercepts are different across the two nations. Additionally, we found full cross-informant invariance, which means that the instrument works invariantly across child and mother when they report about receiving support from each other.

**Table 4:** Cross-national and cross-informant invariance of the similarity model for the received support within the mother–child relationship.

**Model**	**χ^2^**	***P***	***df***	**RMSEA**	**RMSEA (90% CI)**	**CFI**	**SRMR**	**Δχ^2^**	***Δdf***	***p***	**ΔCFI**	**ΔRMSEA**
Configural	773.18	<0.001	250	0.086	(0.079 0.093)	0.930	0.044					
**Cross-national invariance**												
Metric	877.64	<0.001	266	0.090	(0.084 0.097)	0.918	0.081	104.46	16	<0.001	−0.012	0.004
Unconstrained to be equal: RCM06	857.24	<0.001	265	0.089	(0.083 0.096)	0.921	0.074	84.06	15	<0.001	−0.009	0.003
Scalar	1000.57	<0.001	280	0.096	(0.089 0.102)	0.903	0.090	122.93	14	<0.001	−0.015	0.006
Unconstrained to be equal: RMC06	952.47	<0.001	279	0.093	(0.086 0.099)	0.910	0.084	74.83	13	<0.001	−0.008	0.003
Strict	990.74	<0.001	295	0.092	(0.085 0.098)	0.907	0.076	38.27	16	0.0014	−0.003	−0.001
**Cross-informant invariance**												
Metric	991.03	<0.001	303	0.090	(0.084 0.096)	0.908	0.076	0.298	8	1.00	0.001	−0.002
Scalar	1066.56	<0.001	312	0.093	(0.087 0.099)	0.899	0.081	75.53	9	<0.001	−0.009	0.003
Strict	1110.93	<0.001	322	0.093	(0.087 0.099)	0.894	0.087	44.36	10	<0.001	−0.005	0.000

*χ^2^, chi-square test; df, degree of freedom; RMSEA, root mean square error of approximation; CI, confidence interval; CFI, comparative fit index; SRMR, standardized root mean square residual. For each released item: R, received support; MC, mother reports on child; CM, child reports on mother; 01–09, item's number, thus, for example, RCM06 means item 6 of received support when child reports on mother*.

Regarding the father–child relationship, we found cross-national partial scalar invariance for both given and received support, cross-informant partial scalar invariance for the given support, and full cross-informant invariance for the received support (see Supplementary Material).

#### Cross-National and Cross-Informant Measurement Invariance Within LCM (Aim 3)

We tested two multigroup LCMs separately for *similarity in given support* and *similarity in received support*, one without considering measurement invariant constraints, and the other adding the cross-national and cross-informant measurement invariance constraints that we found to be plausible in the aim 2 section. As we aimed to verify if LCM second-order factors (LEVEL and L_SIM) had equivalent variances and means across the two nations, we first run a multi-group model (configural) in which second-order factors were free to vary across the two nations. As reported in Table 5, all these parameters were freely estimated, except for LEVEL of the first group (Germany) and means of L_SIM, which were fixed to zero in order to make the model identified. The configural LCMs for both given and received support had sufficient fit indices (Table 6). We then proceeded testing variance equivalence of the factors across the nations. In other words, we constrained the variance of the second-order factors in the German group to be equivalent to the corresponding second-order factors in the Italian group. Fit indices of these models were not significantly different from those of the unconstrained models, thus indicating that the variances of the two second-order factors are equivalent across the two nations for both the given and the received support similarity models. Finally, we tested mean equivalence of the factors fixing the mean of the Italian second-order factors to zero (i.e., constraining them to be equivalent to that of the German). These models were good for both the similarity models, and they were sufficiently similar to the previous ones. Therefore, we can also conclude that the means of the second-order factors are equivalent across the two nations.

**Table 5:** Variances and means of second-order factors for the given and received support similarity models.

	**LCM with no measurement invariance constraints**				**LCM with measurement invariance constraints**			
**Factor**	**Variances**		**Means**		**Variances**		**Means**	
	**Germany**	**Italy**	**Germany**	**Italy**	**Germany**	**Italy**	**Germany**	**Italy**
**Given support**								
LEVEL	0.458	0.385	0	−0.016	0.467	0.389	0	0.115
L_SIM	0.473	0.386	0	0.471	0.497	0.382	0	0.223
LEVEL with L_SIM	GE: −0.082 (*p* = 0.09)		IT: 0.006 (*p* = 0.93)		GE: −0.037 (*p* = 0.22)		IT: −0.028 (*p* = 0.40)	
**Received support**								
LEVEL	0.524	0.511	0	−0.086	0.535	0.541	0	0.005
L_SIM	0.567	0.719	0	0.247	0.621	0.697	0	−0.114
LEVEL with L_SIM	GE: 0.057 (*p* = 0.24)		IT: −0.262 (*p* < 0.001)		GE: −0.015 (*p* = 0.68)		IT: −0.098 (*p* = 0.06)	

*LCM, latent congruence model; L_SIM, lack of similarity factor; GE/IT, Germany/Italy*.

**Table 6:** Cross-national LCM for similarity models within the mother–child relationship.

**Model**	**χ^2^**	***p***	***df***	**RMSEA**	**RMSEA (90% CI)**	**CFI**	**SRMR**	**Δχ^2^**	***Δdf***	***p***	**ΔCFI**	**ΔRMSEA**
**Given support**												
*Without measurement invariance*												
Configural	593.93	<0.001	250	0.070	(0.063 0.077)	0.941	0.050					
Variance equality	595.12	<0.001	252	0.070	(0.062 0.077)	0.941	0.051	1.19	2	0.550	0.000	0.000
Mean equality	611.80	<0.001	254	0.071	(0.064 0.078)	0.939	0.054	16.68	2	<0.001	−0.002	0.001
*With measurement invariance*												
Configural	870.92	<0.001	313	0.080	(0.073 0.086)	0.905	0.102					
Variance equality	875.38	<0.001	315	0.080	(0.073 0.086)	0.904	0.110	4.46	2	0.108	−0.001	0.000
Mean equality	893.98	<0.001	317	0.080	(0.074 0.087)	0.901	0.121	18.60	2	<0.001	−0.003	0.000
**Received support**												
*Without measurement invariance*												
Configural	773.18	<0.001	250	0.086	(0.079 0.093)	0.930	0.044					
Variance equality	775.24	<0.001	252	0.086	(0.079 0.093)	0.930	0.048	2.06	2	0.357	0.000	0.000
Mean equality	781.14	<0.001	254	0.086	(0.079 0.093)	0.929	0.050	5.90	2	0.052	−0.001	0.000
*With measurement invariance*												
Configural	1137.64	<0.001	323	0.095	(0.089 0.101)	0.891	0.091					
Variance equality	1138.20	<0.001	325	0.095	(0.088 0.100)	0.891	0.092	0.55	2	0.758	0.000	0.000
Mean equality	1141.15	<0.001	327	0.094	(0.088 0.100)	0.891	0.093	2.95	2	0.229	0.000	−0.001

*χ^2^, chi-square test; df, degree of freedom; RMSEA, root mean square error of approximation; CI, confidence interval; CFI, comparative fit index; SRMR, standardized root mean square residual*.

We then proceeded testing the same models but including the measurement invariant constraints that we found to be plausible in the aim 2 section (Table 6). As the constrained models were not relevantly different from the not constrained ones, we concluded that both the variances and the means of the two second-order factors for both the given support and the received support are equivalent across Germany and Italy (Table 5). However, it should be noted that the mean of L_SIM in given support could be affected by differences in cross-national and cross-informant intercepts, and the mean of L_SIM in received support could be affected by differences in cross-national intercepts (see aim 2 section). For those reasons, we should be cautious in concluding that mother and child similarly perceive given and received support and that there are no cross-national differences in those perceptions. The same conclusions can be drawn from the father–child relationship, since the means of the second-order factors are equivalent across the two nations for both given and received support (see Supplementary Material).

### Accuracy

#### LCM (Aim 1)

Differently from the similarity, the accuracy of the reports of mother and child is evaluated including in the same model both the reports referring to the given support and the ones referring to the received support from both informants, because in this way it is possible to test accuracy in both downward and upward support at the same time. Each LCM was run separately for each nation, and we imposed cross-informant constraints for mother and child reports when the two informants were evaluating the same support exchange (e.g., equivalence between the support given by the mother and received by the child).

The model, run on the German sample, had good fit indices [χ^2^(586) = 1323.29; *p* < 0.001; RMSEA = 0.058 (0.053 0.062), *p* = 0.001; CFI = 0.93; SRMR = 0.076] and indicated that the lack of accuracy for the downward support exchange (support given by the mother and received by the child) was on average −0.045 (*p* = 0.216) and its variance was 0.379 (*p* < 0.001), while the variance of the LEVEL_MC factor was 0.457 (*p* < 0.001). Instead, the upward support exchange (support given by the child and received by the mother) was 0.087 (*p* = 0.055), and its variance was 0.574 (*p* < 0.001), while the variance of the LEVEL_CM factor was 0.528 (*p* < 0.001).

The same model for the Italian sample obtained worse fit indices because of the smaller sample size [χ^2^(586) = 1300.37; *p* < 0.001; RMSEA = 0.082 (0.076 0.088), *p* < 0.001; CFI = 0.871; SRMR = 0.094]. In this nation, the lack of accuracy was on average 0.128 (*p* = 0.066) [variance = 0.725 (*p* < 0.001)], and the variance of the LEVEL_MC factor was 0.528 (*p* < 0.001) for the downward support exchange; while the lack of accuracy was on average −0.037 (*p* = 0.474) [variance = 0.366 (*p* < 0.001)], and the variance of the LEVEL_MC factor was 0.353 (*p* < 0.001) for the upward support exchange.

We can conclude that in both nations there is high accuracy in the evaluation of upward and downward support exchanges between mother and child, as the lack of accuracy between the two informants always showed non-significant estimates. Accuracy models referred to the father–child relationship showed that, in both nations, father perceives to give more support than the support received by child (downward support exchange). Regarding the upward exchange, accuracy was found in Italy, while in Germany the child perceives to give less support than the father perceives to receive (see Supplementary Material).

#### Cross-National and Cross-Informant Invariance (Aim 2)

As for the similarity models, we run a multi-group model and fixed to 0 the means of all factors and to 1 the variances of all factors of the first group (Germany), while we freely estimated factor loadings of both groups and means and variances of factors of the second group (Italy). As this configural model presented good model fit indices (see Table 7), we proceeded testing cross-national measurement invariance. For all the four first-order factors (support given by the mother, received by the child, given by the child, received by the mother) included in the model, we found that their items had equivalent factor loadings across nations (full metric invariance). Instead, regarding scalar invariance, we found that item 6 (“Offered to conduct a conversation”) worked differently across nations for each of the four factors. In particular, these were intercepts of item 6 (Germany vs. Italy) when the item was reported by the child, referring to received (3.37 vs. 4.09) or given (3.28 vs. 4.10) support to the mother, and when this item was reported by the mother, referring to received (3.23 vs. 3.67) or given (3.43 vs. 3.80) support to the child. In other words, Italians tend to score higher than Germans. Tested residuals (all except residual of item 6) were equivalent across nations. As for each factor eight out of the nine items were fully invariant across nations, we concluded that each factor has the same meaning within the two nations (metric cross-national invariance) and that their latent (scalar cross-national invariance) and observed (strict cross-national invariance) total scores can be compared across groups.

**Table 7:** Cross-national and cross-informant invariance of the accuracy model for the mother–child relationship.

**Model**	**χ^2^**	***p***	***df***	**RMSEA**	**RMSEA (90% CI)**	**CFI**	**SRMR**	**Δχ^2^**	***Δdf***	***p***	**ΔCFI**	**ΔRMSEA**
Configural	2158.48	<0.001	1068	0.060	(0.057 0.064)	0.932	0.061					
**Cross-national invariance**												
Metric	2305.44	<0.001	1100	0.062	(0.059 0.066)	0.925	0.075	146.96	32	<0.001	−0.007	0.002
Scalar	2644.85	<0.001	1132	0.069	(0.065 0.072)	0.906	0.094	339.41	32	<0.001	−0.019	0.007
Unconstrained to be equal: GCM06, RCM06, RMC06, GMC06	2485.66	<0.001	1128	0.065	(0.062 0.069)	0.915	0.080	180.22	28	<0.001	−0.010	−0.004
Strict	2553.04	<0.001	1160	0.065	(0.062 0.069)	0.913	0.079	67.38	32	0.001	−0.002	0.000
**Cross-informant invariance**												
Metric	2602.34	<0.001	1176	0.066	(0.062 0.069)	0.911	0.082	49.31	16	<0.001	−0.002	0.001
Scalar	2760.23	<0.001	1194	0.068	(0.065 0.072)	0.902	0.086	157.89	18	<0.001	−0.009	0.002
Strict	2931.12	<0.001	1214	0.071	(0.068 0.074)	0.893	0.093	170.89	20	<0.001	−0.009	0.003

*χ^2^, chi-square test; df, degree of freedom; RMSEA, Root Mean Square Error of Approximation; CI, Confidence Interval; CFI, Comparative Fit Index; SRMR, Standardized Root Mean Square Residual; for each released item: G, given support; R, received support; MC, mother reports on child; CM, child reports on mother; 01–09, item's number, thus, for example GCM06 means item 6 of given support when child reports on mother*.

Maintaining in the model the cross-national invariance constraints, we proceed testing the measurement invariance across informant. In other words, we verified if the instrument works equivalently across the two informants that are reporting about both downward and upward support exchanges. We found that all the nine items were fully invariant across the two informants, concluding that mother and child interpret in the same way the items when referring to both downward and upward support exchanges, and that their latent and observed scores are comparable. Regarding the father–child relationship, we found the same results: cross-national partial scalar invariance and full cross-informant invariance for all the accuracy models (see Supplementary Material).

#### Cross-National and Cross-Informant Measurement Invariance Within LCM (Aim 3)

As happened with similarity models, we first run a multi-group model in which second-order factors were free to vary across the two nations, and in which no measurement invariance constraints were added. As reported in Table 8, all these parameters are freely estimated, except for the means of the first group (Germany), which were fixed to zero in order to make the model identified. This LCM had good fit indices (Table 9), so we proceeded testing the variance equivalence of the factors across nations. This model had good fit indices, which were not relevantly different from those of the unconstrained model, thus indicating that the variances of the four second-order factors are equivalent across the two nations. Finally, we tested the mean equivalence of the factors (Table 9), and the model was sufficiently similar to the previous one, to conclude that the means of the four second-order factors are equivalent across the two nations.

**Table 8:** Variances and means of second-order factors for the accuracy models.

	**LCM with no measurement invariance constraints**				**LCM with measurement invariance constraints**			
**Factor**	**Variances**		**Means**		**Variances**		**Means**	
	**Germany**	**Italy**	**Germany**	**Italy**	**Germany**	**Italy**	**Germany**	**Italy**
LEVEL_MC	0.457	0.533	0	0.010	0.504	0.422	0	0.040
L_ACC_MC	0.375	0.731	0	0.437	0.419	0.602	0	0.121
LEVEL with L_ACC_MC	GE: −0.041 (*p* = 0.023)		IT: −0.117 (*p* = 0.002)		GE: −0.054 (*p* < 0.001)		IT: −0.091 (*p* < 0.001)	
LEVEL_CM	0.533	0.345	0	−0.106	0.491	0.422	0	0.011
L_ACC_CM	0.577	0.358	0	−0.303	0.544	0.436	0	−0.033
LEVEL with L_ACC_CM	GE: −0.014 (*p* = 0.565)		IT: −0.038 (*p* = 0.103)		GE: −0.017 (*p* = 0.286)		IT: −0.048 (*p* = 0.013)	

*LCM, latent congruence model; L_ACC, lack of accuracy factor, GE/IT, Germany/Italy*.

**Table 9:** Cross-national LCM for accuracy model within the mother–child relationship.

**Model**	**χ^2^**	***p***	***df***	**RMSEA**	**RMSEA (90% CI)**	**CFI**	**SRMR**	**Δχ^2^**	***Δdf***	***p***	**ΔCFI**	**ΔRMSEA**
**Without measurement invariance**												
Configural	2163.29	<0.001	1072	0.060	(0.056 0.064)	0.932	0.064					
Variance equality	2187.99	<0.001	1076	0.061	(0.057 0.064)	0.931	0.069	24.70	4	<0.001	−0.001	0.001
Mean equality	2210.18	<0.001	1080	0.061	(0.057 0.065)	0.929	0.072	22.19	4	<0.001	−0.002	0.000
**With measurement invariance**												
Configural	2939.16	<0.001	1220	0.071	(0.067 0.074)	0.893	0.094					
Variance equality	2950.75	<0.001	1224	0.071	(0.067 0.074)	0.892	0.097	11.60	4	0.021	−0.001	0.000
Mean equality	2957.39	<0.001	1228	0.071	(0.067 0.074)	0.892	0.098	6.63	4	0.157	0.000	0.000

*χ^2^, chi-square test; df, degree of freedom; RMSEA, root mean square error of approximation; CI, confidence interval; CFI, comparative fit index; SRMR, standardized root mean square residual*.

A second multi-group LCM was tested, following the same procedure just described, but including the measurement invariant constraints that we found to be plausible in the aim 2 section. Also, in this case, we concluded that both the variances and the means of the four second-order factors are equivalent across Germany and Italy (Table 9). The same conclusion can be drawn for the father–child relationship (see Supplementary Material).

## Discussion

In this study, we illustrated how to conduct a cross-national comparison of parent–child similarity and accuracy in support exchanges perceptions applying LCM. The illustrative example shows that LCM is a useful model able to keep into account, at the same time, cross-informant and cross-national measurement invariance when investigating and interpreting parent–child similarity and accuracy. To the knowledge of the authors, no previous study compared parent–child similarity and accuracy between two or more nations. Thus, this is the first study that deals with those comparisons, accepting the challenge to test both cross-informant and cross-national measurement invariance in the same LCM.

An issue that we needed to keep into consideration was deciding the sequence of models tested. Usually, LCM is tested first verifying the configural cross-informant measurement invariant LCM, and then adding step-by-step constraints referred, respectively, to metric, scalar, and strict invariance (Cheung, 2009). That procedure is fundamental to correctly estimate and interpret the amount parent–child discrepancy found. When a cross-national or cross-cultural research design is used and LCM is applied, two different aspects of measurement invariance are involved. Indeed, data could be affected by differences in measurement models because of cross-national differences, cross-informant differences, or both. It highlights the importance of detecting the kind of measurement (non)invariance within the data, besides the level of measurement (non)invariance (metric, scalar, strict). For those reasons, it is important to first test cross-national measurement invariance and then to add the cross-informant one. To test cross-national measurement invariance, all the measurement invariance levels should be verified within a multigroup approach that compares the measurement models of two nations. After finding the best fitting cross-national measurement invariant LCM, it is possible to start the second step-by-step comparison, checking whether there is equivalence in the measurement models of the two informants.

Findings and scenarios could be very different, we could have full cross-national and full cross-informant invariance, partial cross-national and full cross-informant invariance, full cross-national and partial cross-informant invariance, and partial cross-national and partial cross-informant invariance. Moreover, partial invariance could be at different levels: metric (for instance, we found partial cross-national metric invariance in mother–child similarity in received support), scalar (as we found in most of the models, especially in cross-national comparisons), or strict (for instance, we found partial cross-informant strict invariance in mother–child similarity in given support). Table 10 summarizes those scenarios and indicates full or partial invariance found in the illustrative example.

**Table 10:** Cross-national LCM measurement invariance scenarios and findings on the illustrative data.

			**Mother-child relationship**			**Father-child relationship**		
**Congruence**	**Measurement invariance**	**Support**	**Metric**	**Scalar**	**Strict**	**Metric**	**Scalar**	**Strict**
Similarity	Cross-national	Given	Full	Partial	Full	Full	Partial	Full
		Received	Partial	Partial	Full	Full	Partial	Full
	Cross-informant	Given	Full	Partial	Partial	Full	Partial	Full
		Received	Full	Full	Full	Full	Full	Full
Accuracy	Cross-national		Full	Partial	Full	Full	Partial	Full
	Cross-informant		Full	Full	Full	Full	Full	Full

The most desirable situation is the one in which both full cross-national and full cross-informant measurement invariance are found. In that case, researchers are sure that they can interpret the second order variable of the LCM (lack of similarity or lack of accuracy) as due to the level differences in the perception of the evaluated construct. However, very often, real data, as the ones presented in this study, reveal partial measurement invariance at some level.

Finding partial metric invariance in cross-national comparison questions the meaning of the construct in the different nations, and separately for each informant; thus, when similarity and accuracy are compared across nations, it is important to consider that the meaning of the construct on which similarity and accuracy are evaluated is slightly different in the two (or more) nations. Partial scalar invariance in cross-national comparison strongly affects the interpretation of similarity and accuracy and the cross-national comparison, and this is even more problematic if cross-national partial scalar invariance is found for only one informant. Similarly, when cross-informant partial metric invariance is found, the researcher is unsure about the interpretation of the similarity or accuracy variable, because he/she is not sure that the meaning of the construct is equivalent for the two informants. Also, cross-informant partial scalar invariance could affect the amount of similarity and accuracy, because one of the two informants partially overestimates (or underestimates) the level of the construct, so the discrepancy could be affected. Partial strict invariance is the less problematic situation, because the amount of residual is not directly involved in the estimation of similarity and accuracy latent variables. Generally speaking, if partial invariance is due to only one parameter, this reasonably does not have a great impact on the interpretation of results (Dimitrov, 2010), but if there are more items involved, researchers need to reflect on the meaning and amount of similarity and accuracy. For instance, in the analyses, many problems are related to item 6 (“Offered to conduct a conversation”): many cross-national measurement differences are due to the fact that support scores of Italian parents and children could be overestimated, because the intercept of that item is higher compared with the parameter for German parents and children. One possible reason for that could be the higher percentage of Italian young adults cohabiting with their parents than German ones. Since that finding is pervasive, researchers could evaluate to delete the item from analyses. In other cases, it could be sufficient to be aware of possible biases in the estimates.

LCM and the techniques we have used here can also be applied to other types of discrepancies, not only to cross-informant discrepancy, for instance, discrepancy between real and ideal personal characteristics (Liu et al., 2005), or between ideal and actual partner (Knee et al., 2001), or between received and desired support (Wang, 2019), and so on.

This study focused on the application of LCM to just one construct adopting a cross-national dyadic design. In the illustrative example, the construct was emotional support, and we found differences in similarity and accuracy (for instance, especially for given support, parent–child dyads are characterized by less similarity and more accuracy). Those findings revealed that, for parent–child emotional support exchanges literature, it would be important to consider, at the same time, similarity and accuracy processes especially when family triads are studied. Considering similarity and accuracy in the different family dyads allows to adopt a family research approach (Lanz et al., 2015). However, in order to understand the meaning of similarity and accuracy referred to a specific construct, it would be important to also consider other constructs as validity criterion (for some examples see Al Ghriwati et al., 2018; Makol et al., 2019).

Future research could use LCM as a more general framework to investigate complex models, in which validity criteria and predictors and outcomes of congruence (discrepancy, similarity or accuracy) are included (Cheung, 2009). However, Edwards (2009) criticized LCM because, although it constitutes advancement from a measurement point of view (i.e., LCM allows estimating measurement errors and measurement invariance), it presents the same problems as dyadic indexes. Dyadic indexes of discrepancy do not consider the contribution of the individual level (Rogers et al., 2018). That is not a problem *per se*, but it can undermine the interpretation of findings when dyadic indexes are used as predictors or outcomes in more complex models. Indeed, main effects are confounded with interaction/moderation effect and interpretation of findings can be misleading (Rogers et al., 2018; Laird, 2020). According to Edwards (2009), LCM focuses on level and congruence second-order factors defining them as different from the components, but it is not able to solve some problems of interpretations because of the fact that mean or different scores used as predictors or outcomes could hide the main effects of the components.

Literature presents many solutions to those problems. For instance, regression-based approaches, such as polynomial regression models (Edwards, 1994; Laird and Weems, 2011; Laird and De Los Reyes, 2013) or response surface analysis (Edwards, 1994; Barranti et al., 2017; RSA), allow to control the individual level (main effects) to test discrepancy (interaction effect) by including the component measures (individual level), their interaction (dyadic level), and higher-order terms as independent variables in the equation. Also, the Truth and Bias model (T and B; West and Kenny, 2011; Stern and West, 2018) considers both individual and dyadic processes, and allows testing, at the same time, two types of accuracy (i.e., mean-level bias and correlational accuracy). Finally, research questions dealing with interdependent outcomes (e.g., relationship satisfaction of both partners) can be answered by the Actor-Partner Interdependent Model (APIM) framework, which allows considering at the same time both predictors and outcomes measured by the two partners of the dyad; for instance, Schönbrodt et al. (2018) proposed the Dyadic Response Surface Analysis (DRSA), which integrates RSA with APIM and allows testing similarity effect on each of the outcome of two partners.

However, most of those solutions do not allow dealing with measurement model, that, as shown in this study, can be important to reflect on how the construct is measured and which items are more different across nations and/or informants. Recently, some models have been proposed, which allow keeping into consideration measurement models and also, potentially, to deal with measurement invariance issues. One of those models is Latent Moderated Structural Equations (Su et al., 2019), which extends the strengths of polynomial regression models to a SEM in which measurement invariance can be tested. Moreover, as happened for polynomial regression models, it allows testing not only linear relationships among predictors and outcomes but also curvilinear relationships that, for some theoretical frameworks, could be more interesting (Cheung, 2009; Edwards, 2009). However, Su et al. (2019) did not discuss the impact of measurement cross-informants (non)invariance on findings.

This study shows the importance of considering cross-informant and cross-national measurement (non)invariance in research on parent–child discrepancy, similarity, accuracy, and congruence. In that sense, LCM (both regarding similarity and accuracy) and the procedure we propose here are useful when the interest of research is to understand if parent–child discrepancy is similar or different across groups (nations, countries, age groups, and so on). On the opposite, when the research aim is to test predictive relationships between discrepancy and outcomes in different groups, other models, such as Latent Moderated Structural Equations, could be used, but they need to be adapted in order to consider both cross-informant and cross-group invariance. Overall, solid knowledge of conceptual and psychometric frameworks of methods used to measure dyadic similarity and accuracy is fundamental to choose the most appropriate technique to prevent researchers from erroneous or incomplete interpretations of their data.

## Data Availability Statement

The raw data supporting the conclusions of this article will be made available by the authors, without undue reservation.

## Ethics Statement

Ethical review and approval was not required for the study on human participants in accordance with the local legislation and institutional requirements. The patients/participants provided their written informed consent to participate in this study.

## Author Contributions

ST conceived the idea for the paper, contributed to the design of the study, was involved in all steps of the research process, and wrote a first set-up and draft of the manuscript. MZ and AS conducted and interpreted statistical analyses and drafted and edited the manuscript. SS, CH, HB, and ML contributed to the design of the study, data collection and adjustments, and wrote additions. ML made a substantial, direct and intellectual contribution to the work. All authors approved the manuscript and agreed to be accountable for all aspects of the work.

## Conflict of Interest

The authors declare that the research was conducted in the absence of any commercial or financial relationships that could be construed as a potential conflict of interest.

## Publisher's Note

All claims expressed in this article are solely those of the authors and do not necessarily represent those of their affiliated organizations, or those of the publisher, the editors and the reviewers. Any product that may be evaluated in this article, or claim that may be made by its manufacturer, is not guaranteed or endorsed by the publisher.

## Footnotes

^1^Thönnissen, C., Wilhelm, B., Fiedrich, S., Alt, P., and Walper, S. (2015). *Pairfam Scales Manual. Wave 1 to 6*. Unpublished.

^2^All the parameters reported in this paper are non-standardized estimates.
