# Supplementary material for: Latent Congruence Model to Investigate Similarity and Accuracy in Family Members' Perception: The Challenge of Cross-National and Cross-Informant Measurement (Non)Invariance
Source: Front Psychol. 2021 Aug 11;12:672383. doi: 10.3389/fpsyg.2021.672383 (PMC8385144; doi:10.3389/fpsyg.2021.672383)
Supplement: Supplementary file 1 [file Data_Sheet_1.docx]

**Appendix A**

*Child-version of the instrument*

*“In the following it is questioned if and how parents and their children support each other mutually. Please indicate: which kind of assistance did you give to your parents in the last 12 months? / which kind of assistance did you receive form your parents in the last 12 months?”*

Answering Scale: rarely (1) - not very often (2) - occasionally (3) - somewhat often (4) - very often (5)

1. Advice regarding personal problems*.

2. Comfort.

3. Conducted conversations concerning my parent’s personal topics.

4. Conducted conversations concerning my parent’s daily issues.

5. Listened when there were problems.

6. Offered to conduct a conversation.

7. Uttered encouragement.

8. Talk to you about your worries and troubles*.

9. A hug.

* Items from Thönnissen, C., Wilhelm, B., Fiedrich, S., Alt, P. & Walper, S. (2015). Pairfam Scales Manual. Wave 1 to 6. Unpublished.

**Appendix B**

In the table below, the 15 steps of the analyses are described. For each step, there is the description and the tests used to evaluate it. The 15 steps should be done separately for similarity and accuracy models and for each dyad.

| Order | Description of the step | Evaluation |
| --- | --- | --- |
| 1 | Testing LCM separately for each nation. | Fit indexes |
| 2 | **Cross-national** multigroup of first order model (dyadic CFA) – configural model | Fit indexes |
| 3 | **Cross-national** multigroup of first order model (dyadic CFA) – metric model | ${\Delta\chi}^{2}$ ΔCFI ΔRMSEA |
| 4 | **Cross-national** multigroup of first order model (dyadic CFA) – strong model | ${\Delta\chi}^{2}$ ΔCFI ΔRMSEA |
| 5 | **Cross-national** multigroup of first order model (dyadic CFA) – strict model | ${\Delta\chi}^{2}$ ΔCFI ΔRMSEA |
| 6 | **Cross-informant** multigroup of first order model (dyadic CFA) – configural model (maintainig the constraints of the cross-national multigroup model) | Fit indexes |
| 7 | **Cross-informant** multigroup of first order model (dyadic CFA) – metric model (with the constraints of the cross-national multigroup model) | ${\Delta\chi}^{2}$ ΔCFI ΔRMSEA |
| 8 | **Cross-informant** multigroup of first order model (dyadic CFA) – strong model (with the constraints of the cross-national multigroup model) | ${\Delta\chi}^{2}$ ΔCFI ΔRMSEA |
| 9 | **Cross-informant** multigroup of first order model (dyadic CFA) – strict model (with the constraints of the cross-national multigroup model) | ${\Delta\chi}^{2}$ ΔCFI ΔRMSEA |
| 10 | **Cross-national** multigroup LCM without measurement or structural constraints | Fit indexes |
| 11 | **Cross-national** multigoup LCM without measurement constraints but adding factors’ variance equivalence across nations | ${\Delta\chi}^{2}$ ΔCFI ΔRMSEA |
| 12 | **Cross-national** multigoup LCM without measurement constraints but adding factors’ variance and factors‘ mean equivalence across nations | ${\Delta\chi}^{2}$ ΔCFI ΔRMSEA |
| 13 | **Cross-national** multigroup LCM with measurement invariant constraints found in steps 2 to 9 | Fit indexes |
| 14 | **Cross-national** multrigoup LCM with measurement invariant constraints found in steps 2 to 9 and adding factors’ variance equivalence across nations | ${\Delta\chi}^{2}$ ΔCFI ΔRMSEA |
| 15 | **Cross-national** multrigoup LCM with measurement invariant constraints found in steps 2 to 9 and adding factors’ variance and factors‘ mean equivalence across nations | ${\Delta\chi}^{2}$ ΔCFI ΔRMSEA |
