# Supplementary material for: Latent Congruence Model to Investigate Similarity and Accuracy in Family Members' Perception: The Challenge of Cross-National and Cross-Informant Measurement (Non)Invariance
Source: Front Psychol. 2021 Aug 11;12:672383. doi: 10.3389/fpsyg.2021.672383 (PMC8385144; doi:10.3389/fpsyg.2021.672383)
Supplement: Supplementary file 2 [file Data_Sheet_2.docx]

**Supplementary materials of “Latent congruence model to investigate similarity and accuracy in family members’ perception: the challenge of cross-national and cross-informant measurement (non)invariance.”**

**Reliabilities**

| Table S1.  *Estimation of the composite reliability (ω) for each version of the scale separately for the two countries* | | |
| --- | --- | --- |
| Emotional support… | Germany | Italy |
| given from the child to the mother | .909 | .902 |
| given from the child to the father | .911 | .908 |
| received by the child from the mother | .928 | .948 |
| received by the child from the father | .942 | .948 |
| given from the mother to the child | .927 | .922 |
| given from the father to the child | .927 | .921 |
| received by the mother from the child | .939 | .938 |
| received by the father from the child | .941 | .935 |

**Results for the father-child relationship**

**Similarity**

***LCM (aim 1)***

We followed exactly the procedure described for the mother-child relationship. We run separate for Germany and Italy two LCMs, one for each support direction (given/received). In each model we imposed cross-informant constraints and we estimate the amount of (lack of) similarity between the two informants.

Both models (given and received support similarity modes) run on the Germany sample had sufficient fit indices [given support: χ2 (150) = 590.18; p<.001; RMSEA = .088 (.081 .096); CFI = .889; SRMR = .075; received support: χ2 (150) = 439.01; p<.001; RMSEA = .072 (.064 .079); CFI = .941; SRMR = .044]. The lack of similarity for the given support between father and child was -.041 (p=.181) [variance = .294 (p<.001)], and the variance of the LEVEL factor was .479 (p<.001), while for the received support the lack of similarity was -.006 (p=.905) [variance = .759 (p<.001)], and the variance of the LEVEL factor was .597 (p<.001).

The same models were run for the Italian sample obtaining worse fit indices due to the smaller sample size for both models [given support: χ2 (150) = 426.35; p<.001; RMSEA = .100 (.089 .112); CFI = .842; SRMR = .098; received support: χ2 (150) = 398.98; p<.001; RMSEA = .095 (.084 .107); CFI = .897; SRMR = .070]. In this nation the lack of similarity for the given support was on average .272 (p <.001) [variance = .567 (p<.001)], and the variance of the LEVEL factor was .429 (p<.001), while for the received support the lack of similarity was -.032 (p =.710) [variance = 1.043 (p<.001)] and the variance of the LEVEL factor was .559 (p<.001).

These results showed that, only in Italy, father perceives to give more support than child. On the contrary, similarity in received support (both nations) and similarity in given support (only Germany) was found in father-child dyads.

***Cross-national and cross-informant invariance (aim 2)***

We tested the cross-national measurement invariance for both the *given support similarity model* and the *received support similarity model.*

Regarding the *given support similarity model*, we found a partial scalar invariance as item 6 (“Offered to conduct a conversation”) and item 3 (“Conducted conversations concerning my child/father personal topics”) worked differently across nations. In particular, item 3 had higher intercepts in Germany than in Italy both when the informant was the father (3.23 vs. 2.997) as well as the child (3.15 vs. 2.70), and item 6 showed higher intercepts in Italy than in Germany both when the informant was the father (3.37 vs. 3.02), as well as the child (3.59 vs. 2.73).

Furthermore, we tested cross-informant invariance (see Table S1-S2) finding a partial scalar invariance, as item 3’s and item 4’s (“Conducted conversations concerning my child/father daily issues”) intercepts differed across informants for the German sample, while item 6’s intercepts differed across informants in the Italian sample. Specifically, German children tend to score higher both item 3 (3.15 vs. 2.87) and item 4 (3.66 vs. 3.17) respect to their fathers, and Italian children tend to score higher on item 6 (3.56 vs. 3.02) respect to their fathers.

Regarding the *received support similarity model,* we found a partial scalar invariance, as the intercept of item 6 (“Offered to conduct a conversation”) was higher for Italy both when the child reported to receive support from the father (3.44 vs. 2.76), as well as when the father reported to receive support from the child (3.37 vs. 2.80); moreover, the intercepts of item 3 (“Conducted conversations concerning my parents personal topics”) and item 9 (“Hugged them/Have been hugged by them”) were higher for Germany when the child reported to receive support from the father (item 3’s intercepts 3.17 vs. 2.79; item 9’s intercepts 3.04 vs. 2.66).

Finally, we tested cross-informant invariance finding that the instrument works invariantly across child and father when they report about receiving support from each other (e.g., support received by the child to the father and support received by the father to the child).

***Cross-national and cross-informant measurement invariance within Latent congruence model (aim 3)***

We compared the mean and variance of the second-order factors of the similarity models in two ways. In the first case, we did not include any invariance constraints in the model, while in the second case we included both the cross-nation and cross-informant constraints that were found plausible in the Aim 2.

Regarding the models without invariance constraints, we firstly run a configural model for both given and received support similarity models (see respectively Table S1 and S2) from which we derived the values of second-order factors’ variance and means when these were free to vary across Germany and Italy (see Table S3).

We verified that models in which second-order factors’ variances were constrained to be equivalent across nations were not relevantly different from the configural models. At the same way, we verified that the models in which second-factors’ means were constrained to be equivalent across nations were good and sufficiently similar to the previous ones. We concluded that, when tested without imposing any measurement invariance constraints, second-order factors’ variance and means are equivalent across Germany and Italy.

We repeated the same steps but including in each model the cross-national and cross-informant invariance constraints imposed in the “Aim 2” section. The free variances and means of the configural model are reported in Table S3. Second-order factors’ variances and means were found to be equivalent across Germany and Italy for both given and similarity models.

**Accuracy**

***LCM (aim 1)***

In this aim as well as in the followings, we followed exactly the procedure described for the mother child-relationship. We firstly run, only on the Germany sample, an accuracy LCM including cross-informant and obtained a model with good fit indices [χ2 (586)= 1307.85; p<.001; RMSEA = .057 (.053 .061), p = .003; CFI = .932; SRMR = .062]. This model’s results indicated that the lack of accuracy for the father-child downward support exchange (support given by the father and received by the child) was on average .154 (p = .001) [variance = .566 (p <.001)], while the variance of the LEVEL_FC factor was .554 (p<.001). For the father-child upward exchange (support given by the child and received by the father) the lack of accuracy was on average -.145 (p = .001) [variance = .548 (p <.001)], while the variance of the LEVEL_CF was .525 (p<.001).

When the same model was run for the Italian sample, we obtained worse fit indices due to the smaller sample size [χ2 (586) = 1177.825; p<.001; RMSEA = .074 (.068 .080), p<.001; CFI = 0.883; SRMR= 0.082]. In this model, the lack of accuracy for the downward support exchange was on average .173 (p = .034) [variance = .944 (p <.001)], while the variance of the LEVEL_FC factor was .570 (p<.001). For the upward exchange, the lack of accuracy was on average -.075 (p = .269) [variance = .619 (p <.001)], while the variance of the LEVEL_CF was .369 (p<.001).

***Cross-national and cross-informant invariance (aim 2)***

When testing the cross-national measurement invariance of the four first-order factors referring to the father-child relationship, we found a sufficient level of cross-national invariance (see Table S4) as only the intercept of item 6 (“Offered to conduct a conversation”) was higher in Italy than Germany for each of the four factors. In particular, these were item 6’s intercept (Germany vs. Italy) when this item was reported by the child, referring to received (2.78 vs. 3.51) or given (2.23 vs. 3.63) support, as well as when this item was reported by the father, referring to received (2.82 vs. 3.36) or given (3.01 vs 3.40) support.

Furthermore, we tested cross-informant invariance (see Table S4) finding that the instrument works invariantly across child and father when they report about the same support exchange (i.e., support given by the father and received by the child as well as support given by the child and received by the father).

***Cross-national and cross-informant measurement invariance within Latent congruence model (aim 3)***

Finally, we compared the mean and variance of the second-order factors of the accuracy models in two ways. In the first case, we took do not include any invariance constraints in the model, while in the second case we included both the cross-nation and cross-informant constraints that were found plausible in the aim 2.

Regarding the models without invariance constraints, we firstly run a configural model [$\chi^{2}$ (1072) = 2120.59; p<.001; RMSEA = .059 (.055 .063); CFI = .933; SRMR = .053] from which we derived the values of second-order factors’ variance and means when these are free to vary across Germany and Italy (see Table S5).

We then verified that the model in which second-order factors’ variances were constrained to be equivalent across nations [$\chi^{2}$ (1076) = 2131.90; p<.001; RMSEA = .059 (.055 .063); CFI = .933; SRMR = .067] was not relevantly different from the configural model [${\Delta\chi}^{2}$ (4) = 11.31; p = .02; ΔRMSEA = .000; ΔCFI = .000]. At the same way, we verified that the model in which second-factors’ means were constrained to be equivalent across nations was good [$\chi^{2}$ (1080) = 2139.66; p<.001; RMSEA = .059 (.055 .063); CFI = .933; SRMR = .067] and sufficiently similar to the previous one [${\Delta\chi}^{2}$ (4) = 7.76; p = .10; ΔRMSEA = .000; ΔCFI = .000]. We concluded that, when tested without imposing any measurement invariance constraints, second-order factors’ variances and means are equivalent across Germany and Italy.

We then repeated the same steps, but including cross-nation and cross-informant invariance constraints in the model. In particular, we firstly tested a configural model where items of first-order factors were constrained to be equivalent across nation and informant (i.e. maintaining the constraint imposed in “Aim 2” section) and second-order factors were free to have different variances and means across the two nations [$\chi^{2}$(1220) = 2743.33; p<.001; RMSEA = .067 (.063 .070); CFI = .903; SRMR = .081]. The free variances and means of this model are reported in Table S5.

Second, we compared the model where the second-order factors’ variances were constrained to be equivalent across groups [$\chi^{2}$(1224) = 2749.23; p<.001; RMSEA = .067 (.063 .070); CFI = .903; SRMR = .091] with the configural model, finding that they are not significantly different [$\Delta\chi^{2}$(4) = 5.90; p = .21; ΔRMSEA = .000; ΔCFI = .000]. Finally, we tested a third model imposing the factors’ mean equivalence across nations [$\chi^{2}$(1228) = 2760.77; p<.001; RMSEA = .067 (.063 .070); CFI = .903; SRMR = .095] and also this model was not different from the previous one [${\Delta\chi}^{2}$(4) = 11.54; p = .02; ΔRMSEA = .000; ΔCFI = .000]. We concluded that both the variances and the means of the four second-order factors for the father-child relationship are equivalent across Germany and Italy.

| Table S2.  *Cross-cultural and cross-informant invariance of the similarity model for the given support father-child relationship* | | | | | | | | | | | | |
| --- | --- | --- | --- | --- | --- | --- | --- | --- | --- | --- | --- | --- |
| Model | $\chi^{2}$ | *P* | *df* | RMSEA | RMSEA (90%CI) | CFI | SRMR | ${\Delta\chi}^{2}$ | *Δdf* | *p* | ΔCFI | ΔRMSEA |
| Configural | 693.31 | < .001 | 250 | .080 | (.073 .087) | .922 | .053 |  |  |  |  |  |
| *Cross-cultural invariance* | | | | | | | | | | | | |
| Metric | 717.46 | < .001 | 266 | .078 | (.071 .085) | .921 | .060 | 24.15 | 16 | .086 | -.001 | -.002 |
| Scalar | 955.90 | < .001 | 282 | .092 | (.086 .099) | .882 | .098 | 238.44 | 16 | < .001 | -.039 | .014 |
| unconstrained to be equal GCF06, GFC06,  GCF03, GFC03 | 784.49 | < .001 | 278 | .081 | (.074 .087) | .911 | .065 | 67.03 | 12 | < .001 | -.010 | -.011 |
| Strict | 819.91 | < .001 | 292 | .080 | (.074 .087) | .907 | .068 | 35.43 | 14 | .0013 | -.004 | -.001 |
| *Cross-informant invariance* | | | | | | | | | | | | |
| Metric | 833.81 | < .001 | 300 | .080 | (.074 .087) | .906 | .071 | 13.89 | 8 | .084 | -.001 | .000 |
| Scalar | 1032.39 | < .001 | 310 | .091 | (.073 .086) | .873 | .088 | 198.58 | 10 | < .001 | -.033 | .011 |
| unconstrained to be equal GE: GCF04, GCF03, GFC03;  IT: GCF06, GFC06 | 887.89 | < .001 | 307 | .082 | (.078 .090) | .898 | .079 | 54.08 | 7 | < .001 | -.008 | .002 |
| Strict | 2726.01 | < .001 | 315 | .084 | (.076 .088) | .890 | .083 | 52.13 | 8 | < .001 | -.008 | .002 |
| *Note*. $\chi^{2}$ = chi-square test; *df* = degree of freedom; RMSEA = Root Mean Square Error of Approximation; CI = Confidence Interval; CFI = Comparative Fit Index; SRMR = Standardized Root Mean square Residual; for each released item: G=given support, FC=father reports on child, CF=child reports on father, 01-09: item’s number thus, for example GCF06 means item 6 of given support when child reports on father. | | | | | | | | | | | | |

| Table S3.  *Cross-cultural and cross-informant invariance of the similarity model for the received support father-child relationship* | | | | | | | | | | | | |
| --- | --- | --- | --- | --- | --- | --- | --- | --- | --- | --- | --- | --- |
| Model | $\chi^{2}$ | *p* | *df* | RMSEA | RMSEA (90%CI) | CFI | SRMR | ${\Delta\chi}^{2}$ | *Δdf* | *p* | ΔCFI | ΔRMSEA |
| Configural | 693.03 | < .001 | 250 | .080 | (.073 .087) | .940 | .046 |  |  |  |  |  |
| *Cross-cultural invariance* | | | | | | | | | | | | |
| Metric | 723.23 | < .001 | 266 | .078 | (.072 .085) | .938 | .057 | 30.20 | 16 | .017 | -.002 | -.002 |
| Scalar | 965.83 | < .001 | 282 | .093 | (.087 .100) | .907 | .089 | 242.60 | 16 | < .001 | -.031 | .015 |
| - unconstrained to be equal  RCF06, RFC06, RCF03, RCF09 | 795.90 | < .001 | 278 | .082 | (.075 .088) | .929 | .061 | 72.67 | 12 | < .001 | -.009 | .004 |
| Strict | 829.12 | < .001 | 292 | .081 | (.075 .088) | .927 | .062 | 33.22 | 14 | .003 | -.002 | -.001 |
| *Cross-informant invariance* | | | | | | | | | | | | |
| Metric | 840.33 | < .001 | 300 | .080 | (.074 .087) | .926 | .064 | 11.21 | 8 | .190 | -.001 | -.001 |
| Scalar | 906.57 | < .001 | 309 | .083 | (.077 .090) | .919 | .067 | 66.24 | 9 | < .001 | -.007 | .003 |
| Strict | 948.24 | < .001 | 319 | .084 | (.078 .090) | .914 | .069 | 41.67 | 10 | < .001 | -.005 | .001 |
| *Note*. $\chi^{2}$ = chi-square test; *df* = degree of freedom; RMSEA = Root Mean Square Error of Approximation; CI = Confidence Interval; CFI = Comparative Fit Index; SRMR = Standardized Root Mean square Residual; for each released item: R=received support, FC=father reports on child, CF=child reports on father, 01-09: item’s number thus, for example RCF06 means item 6 of received support when child reports on father. | | | | | | | | | | | | |

| Table S4. *Second-order factors’ variances and means for the given and received support similarity models* | | | | | | | | |
| --- | --- | --- | --- | --- | --- | --- | --- | --- |
|  | LCM with no measurement invariance constraints | | | | LCM with measurement invariance constraints | | | |
| Factor | Variances | | Means | | Variances | | Means | |
|  | Germany | Italy | Germany | Italy | Germany | Italy | Germany | Italy |
| *Given support* | | | | | | | | |
| LEVEL | 0.458 | 0.400 | 0 | 0.047 | 0.483 | 0.390 | 0 | 0.183 |
| L_SIM | 0.461 | 0.549 | 0 | 0.185 | 0.596 | 0.522 | 0 | 0.307 |
| LEVEL with L_SIM | GE: -.015 (p=.76) | | IT: .066 (p=.37) | | GE: -.041 (p=.25) | | IT: -.048 (p=.23) | |
| *Received support* | | | | | | | | |
| LEVEL | 0.597 | 0.577 | 0 | -0.049 | 0.604 | 0.504 | 0 | 0.176 |
| L_SIM | 0.761 | 1.040 | 0 | -0.049 | 0.766 | 0.943 | 0 | -0.026 |
| LEVEL with L_SIM | GE: .081 (p=.18) | | IT: -.206 (p=.03) | | GE: -.089 (p=.03) | | IT: -.114 (p=.052) | |

*Note*. LCM = Latent congruence model; L_SIM: lack of similarity factor, GE/IT: Germany/Italy.

| Table S5.  *Cross-cultural and cross-informant invariance of the accuracy model for the father-child relationship* | | | | | | | | | | | | |
| --- | --- | --- | --- | --- | --- | --- | --- | --- | --- | --- | --- | --- |
| Model | $\chi^{2}$ | *p* | *df* | RMSEA | RMSEA (90%CI) | CFI | SRMR | ${\Delta\chi}^{2}$ | *Δdf* | *p* | ΔCFI | ΔRMSEA |
| Configural | 2117.800 | < .001 | 1068 | .059 | (.055 .063) | .933 | .052 |  |  |  |  |  |
| *Cross-cultural invariance* | | | | | | | | | | | | |
| Metric | 2178.49 | < .001 | 1100 | .059 | (.055 .063) | .932 | .056 | 60.69 | 32 | .002 | -.001 | .000 |
| Scalar | 2505.82 | < .001 | 1132 | .066 | (.062 .069) | .913 | .079 | 327.33 | 32 | < .001 | -.019 | .007 |
| - unconstrained to be equal  GCF06, RCF06, RFC06, GFC06 | 2345.27 | < .001 | 1128 | .062 | (.058 .066) | .923 | .060 | 166.78 | 28 | < .001 | -.009 | -.004 |
| Strict | 2413.15 | < .001 | 1160 | .062 | (.059 .065) | .921 | .062 | 67.88 | 32 | .001 | -.002 | .000 |
| *Cross-informant invariance* | | | | | | | | | | | | |
| Metric | 2437.40 | < .001 | 1176 | .062 | (.058 .065) | .920 | .063 | 24.25 | 16 | .084 | -.001 | .000 |
| Scalar | 2599.99 | < .001 | 1194 | .065 | (.061 .068) | .911 | .068 | 162.59 | 18 | < .001 | -.009 | .003 |
| Strict | 2726.01 | < .001 | 1214 | .067 | (.063 .070) | .904 | .072 | 126.01 | 20 | < .001 | -.007 | .002 |
| *Note*. $\chi^{2}$ = chi-square test; *df* = degree of freedom; RMSEA = Root Mean Square Error of Approximation; CI = Confidence Interval; CFI = Comparative Fit Index; SRMR = Standardized Root Mean square Residual for each released item: G = given support, R=received support, FC=father reports on child, CF=child reports on father, 01-09: item’s number thus, for example GCF06 means item 6 of given support when child reports on father. | | | | | | | | | | | | |

| Table S6.  *Second-order factors’ variances and means for the accuracy models* | | | | | | | | |
| --- | --- | --- | --- | --- | --- | --- | --- | --- |
|  | From Aim 1: no measurement invariance | | | | From Aim 3: with measurement invariance | | | |
| Factor | Variances | | Means | | Variances | | Means | |
|  | Germany | Italy | Germany | Italy | Germany | Italy | Germany | Italy |
| LEVEL_FC | 0.566 | 0.563 | 0 | 0.047 | .603 | 0.490 | 0 | 0.134 |
| L_ACC_FC | 0.585 | 0.958 | 0 | 0.196 | .631 | 0.808 | 0 | 0.173 |
| LEVEL_FC with L_ACC_FC | GE: -.096 (p< .001) | | IT: -.150 (p= .001) | | GE: -.091 (p <.001) | | IT: -.113 (p< .001) | |
| LEVEL_CF | 0.512 | 0.356 | 0 | -0.069 | .491 | 0.383 | 0 | 0.138 |
| L_ACC_CF | 0.553 | 0.624 | 0 | 0.051 | .531 | 0.645 | 0 | -0.076 |
| LEVEL_FC with L_ACC_FC | GE: -.072 (p= .002) | | IT: -.086 (p= .008) | | GE: -.050 (p =.001) | | IT: -.064 (p= .002) | |

*Note*. LCM = Latent congruence model; L_ACC: lack of accuracy factor, GE/IT: Germany/Italy.
